# Ethical leadership supports safety voice by increasing risk perception and reducing ethical ambiguity: Evidence from the COVID‐19 pandemic

**DOI:** 10.1111/risa.14053

**Published:** 2022-10-19

**Authors:** M. Selim Cakir, Jamie K. Wardman, Alexander Trautrims

**Affiliations:** ^1^ University of Bristol Business School University of Bristol Bristol UK; ^2^ Nottingham University Business School University of Nottingham Nottingham UK

**Keywords:** COVID‐19, ethical leadership, risk perception, safety voice, workplace safety

## Abstract

Misconduct by business and political leaders during the pandemic is feared to have impacted people's adherence to protective measures that would help to safeguard against the spread of COVID‐19. Addressing this concern, this article theorizes and tests a model linking ethical leadership with workplace risk communication—a practice referred to as ‘safety voice’ in the research literature. Our study, conducted with 511 employees from UK companies, revealed that ethical leadership is positively associated with greater intention to engage in safety voice regarding COVID‐19. We also find that this association is mediated by relations with the perceived health risk of COVID‐19 and ambiguity about ethical decision making in the workplace. These findings therefore underscore the importance of good ethical conduct by leaders for ensuring that health and safety risks are well understood and communicated effectively by organizational members particularly during crises. We discuss the theoretical and practical implications of our study and highlight further opportunities for future research to address the ethical dimensions of leadership, risk management, and organizational risk communication.

## 1 INTRODUCTION

Risk management and communication research has identified that leadership plays a crucial role in encouraging workplace health, safety, and well‐being (Clarke, 2013; Clarke & Ward, 2006; Flin, 1996; Haslam et al., 2020). Research shows that the ‘tone from the top’ adopted by managers can help to create a ‘risk‐aware’ culture because it sets expectations of acceptable risk levels and affirms the importance of behavioral adherence with health and safety measures to broader strategic goals and organizational operations (Braumann et al., 2020; Chen & Hou, 2016; Stollberger et al., 2020). Previous studies have also found that different leadership styles may influence the willingness of employees to communicate about workplace risks—a behavior referred to in the research literature as ‘safety voice’ (Noort et al., 2019). For instance, adopting an ‘open and inclusive’ approach to risk management helps to promote employee trust and encourages workers to speak up about safety concerns, such as by sharing risk information responsively with managers and other colleagues when new hazards emerge or when unforeseen weaknesses in existing safety measures become known (Conchie & Burns, 2008; Noort et al., 2019). Indeed, ‘Talking with workers about working safely during the COVID‐19 pandemic’ is identified amongst six priority health and safety measures in official UK Government guidelines issued to businesses for reducing the risk of spreading the disease (Health & Safety Executive, 2022a).

However, the multifarious threats posed by COVID‐19 have also confronted managers with difficult moral dilemmas because organizational decisions undertaken in response to the pandemic are inevitably fraught with uncertainties, ambiguities, and trade‐offs regarding possible impacts on occupational health, personal finances, and the economic viability of businesses (Balog‐Way & McComas, 2020; Rode & Fischbeck, 2021; Wardman & Löfstedt, 2020). Many “frontline” and “key workers,” whose labor ensures that supplies of vital goods keep circulating and essential services remain open, have for instance been asked to perform their duties often without adequate protective measures in place, which has reportedly resulted in thousands of deaths due to heightened exposure to the virus (Agius, 2020a; Bryce et al., 2020; The Guardian, 2021).

Meanwhile, far from providing the direction and moral resolve required to help people surmount the potent threat presented by COVID‐19, critics argue that pandemic leadership has all too often been marred by a narrow and uncaring outlook, which has served to undermine collective resilience and compromised the safety of individuals and their wider communities (Bryce et al., 2020; Wardman, 2020). In the UK especially, public consternation has followed reports of “non‐essential” businesses intentionally flouting safety rules by continuing to trade illicitly and requiring vulnerable employees to engage in workplace‐based duties (Stevenson, 2021; Trautrims et al., 2020). There have also been reports of numerous lockdown breaches and incidents of illegality in the workplace occurring not only in commercial settings, but also by senior UK government politicians and staff who wrote COVID‐19 rules and regulations covering people's behavior during the pandemic (Reicher, 2021). This has led to charges that "it's one rule for them, and another rule for everyone else," which is feared could undermine the efficacy of public health campaigns requiring collective action and resolve to halt the spread of disease (Reicher et al., 2021; Wardman, 2020).

These issues and concerns therefore raise key questions regarding what impact the moral conduct of leaders might have on people's behavioral intentions to engage in health and safety‐related actions. Yet, while there is often an ethical imperative for the members of an organization to communicate with colleagues about workplace health and safety‐related issues, and the failure to do so can lead to adverse outcomes (Noort et al., 2021), there is little research to date, which specifically investigates how perceptions of a leader's ethical conduct might affect the ways in which risks are interpreted, acted upon, and communicated by other organizational members. Addressing this lacuna, this article presents findings from the first study to our knowledge which investigates how, and by what mechanisms, ethical leadership in organizations impacts the readiness of workers to communicate about health and safety risks. Particularly, our study adds to current understandings by examining the connections between ethical leadership and safety voice and their associations with employee risk perceptions and ethical ambiguity regarding workplace decisions within the context of keeping “COVID‐secure” during the pandemic. We test our model using data drawn from a sample of 511 employees from companies within the United Kingdom during the early phases of the COVID‐19 health crisis. Our findings show that perceptions of ethical leadership are positively associated with the greater intention of workers to engage in safety voice. We also find that this association is mediated by perceived health risk and ethical ambiguity. This study thus contributes to literature through a novel and empirically tested model of leadership and workplace relations that underscores the vital importance of good moral conduct by leaders to effective organizational risk management and communication.

The article proceeds as follows. Section 2 outlines the context of the COVID‐19 global health crisis and highlights concerns that have surfaced regarding the importance of ethical leadership to ensuring workplace safety following reports of poor moral conduct across public and private sectors during the pandemic. Section 3 introduces our conceptual model theorizing the possible associations between ethical leadership, safety voice, risk perception, and ethical ambiguity in the workplace. The basis of the conceptual model is elaborated through a review of the underlying theory and research literature supporting our development of hypotheses. Section 4 presents our research methods, including the sample and variables. Section 5 presents the empirical results. Finally, Section 6 discusses our findings along with the policy implications and study limitations, before concluding with suggested directions for future research.

## 2 ETHICAL LEADERSHIP AND “COVID‐SECURE” WORKPLACES

The COVID‐19 global health crisis has presented organizations with many major challenges and widespread disruptions to normal working practices that have been a great test of their resilience and ability to manage risk (Bryce et al., 2020; Foss, 2020; Wardman & Löfstedt, 2020; Zinn, 2020). Health bodies such as the US Centers for Disease Control (CDC) have identified that workplace settings can be particularly “high risk” due to working conditions which require the close proximity of employees, or that they operate in poorly ventilated areas for prolonged periods (Cunningham et al., 2021; Middleton et al., 2020). In response, many organizations have taken steps to try to ensure that workplaces are “COVID‐secure” in a bid to halt the spread of coronavirus and protect vulnerable employees (Baptista et al., 2021). Improvements to worker safety have included such measures as incorporating physical (social) distancing, the use of personal protective equipment (PPE) such as face masks and gowns, placing transmission barriers and screens at key contact points, regularly disinfecting surfaces, and allowing staff to work from home wherever possible (Liu et al., 2020). As noted above, workplace risk communication about the dangers of the COVID‐19 pandemic has also been encouraged to help raise awareness of the risks and encourage safety behavior (Health and Safety Executive, 2022a). Businesses in places such as the United Kingdom were (until the recent de‐escalation of safety measures) also required by health authorities to report newly discovered cases of COVID‐19 in order to help monitor and quell potential outbreaks (Health and Safety Executive, 2022b).

Alongside these developments, the moral dimensions of COVID‐19 workplace safety and reporting practices have become a salient issue. One widely shared investigative report, “Lost on the Frontline,” conducted by over 100 journalists for Kaiser Health News and the Guardian newspaper found that 3600 US healthcare workers lost their lives during the first year of the pandemic (Spencer & Jewett, 2021). In this report, many preventable deaths were attributed to various factors all seen to contribute to an increased health risk, including insufficient personal protective equipment, confused safety guidance, little notification of health risks by employers to workers, and lax enforcement of health and safety rules by regulators. The investigation also criticized the “hands off” approach to workplace safety taken by the US Labor Department alongside a failure by employers to report COVID‐19‐related worker deaths to the Occupational Safety and Health Administration amidst 4,100 filed safety complaints to regulators and continuing deaths at the workplaces in question (Jewett et al., 2020). Subsequently, a new emergency standard was announced by Labor Department officials to protect health care workers if staying home when sick and alerting their employer about a COVID‐19 hazard, though some “high‐risk” industries were reluctant to share the same emergency rules for controlling the drivers of infection in workplaces (Jewett, 2021).

In the United Kingdom, businesses using the government's centralized Health and Safety Executive (2022b) “RIDDOR” virus reporting system have been credited for demonstrating ethical leadership as “good actors” in the fight against the transmission of the disease. However, alongside this praise reports have surfaced highlighting that new cases of coronavirus contracted in the workplace often go unreported and in some workplace settings cases have been found to be up to 30 times higher than the numbers typically disclosed to authorities (Agius, 2020b; The Guardian, 2020). The owners of well‐known company brands acted in apparent contravention of government safety guidance by insisting that workers return to factories and offices despite national guidelines requiring people to work from home, particularly when ill with COVID‐19 (Agius, 2020a). Subsequently, UK businesses faced accusations of exploiting legal loopholes, faking safety audits, refusing to provide adequate sick pay to those who need to self‐isolate, and demanding that vulnerable employees return to work when sick (Agius, 2020b; Rodgers, 2021; Trautrims et al., 2020). Such behavior led to complaints by workers’ unions that the virus reporting system is a faulty mechanism for ensuring employee safety because it relies too heavily on the “good moral conduct” of employers when this is more often found to be lacking (TUC, 2021). For these reasons, during the course of the pandemic local authorities had to step up their enforcement actions against businesses, and the maximum fixed penalty notice (i.e., fine) for initial rule breaches was increased to £1000 by the UK government, rising to £10,000 following repeated offenses (The Guardian, 2022).

All the while, however, UK government leaders proved susceptible to conduct problems despite having written COVID‐19 legislation and guidance, repeatedly warned of the need for strict observation of the rules, and stated that “every flex can be fatal” (The BBC, 2021). Widely reported incidents brought to light by journalist exposés have ranged across the “Dominic Cummings affair” at the beginning of the first lockdown (Wardman, 2020), the resignation of the Health Secretary Matt Hancock following demands he be dismissed after breaching social distancing guidelines through a tryst at work with his aid (Reicher, 2021), and the “Partygate” scandal engulfing Downing Street and Whitehall offices. In this last series of incidents, senior politicians and government officials—including the then head of the Covid Taskforce, the head of the Civil Service, the Prime Minister Boris Johnson, and the government's then Director General of Propriety and Ethics—belatedly conceded to having attended “social gatherings with drinks” at a time that many forms of social mixing and socializing were prohibited, including that relatives were barred from visiting dying loved ones and tight restrictions limiting attendance at funerals (Reicher, 2021). Following these revelations, a police investigation resulted in more than 50 fixed penalty notices being issued, including to Prime Minister Boris Johnson and Chancellor Rishi Sunak, confirming widespread criminality in government offices (The Guardian, 2022). Concurrently, an internal inquiry into behavioral standards within government—known as the “Sue Gray Report”—highlighted “failures of leadership” as part of its interim findings (Cabinet Office, 2022). In the event, repeated rule breaking has precipitated public anger, accusations of misconduct, and an erosion in the moral authority that would otherwise aid leaders to perform the responsible tasks of setting behavioral guidelines and encouraging wider adherence to COVID‐19 health and safety measures.

## 3 LITERATURE REVIEW AND HYPOTHESIS DEVELOPMENT

**FIGURE 1:**
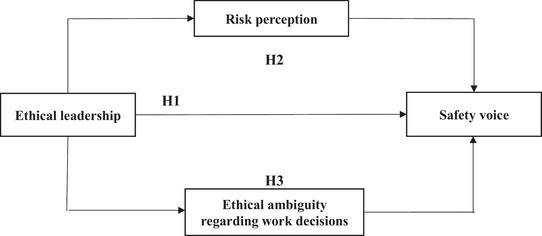
Conceptual model

Safety failures during the pandemic have frequently been associated with rule breaking and lapses in moral conduct by leaders of organizations; it is therefore paramount to examine the role that this might play in ensuring that appropriate health and safety measures are taken against COVID‐19. In this study, we are interested in developing and testing a model which sets out how perceptions of ethical leadership are associated with the intention of employees to engage in safety voice with respect to communicating about COVID‐19 safety and security in the workplace. As COVID‐19 arose as a novel health threat, we are also interested in how these relations are associated with employee health risk perceptions and ethical ambiguity about workplace decisions. Our conceptual model of these proposed associations is presented in Figure 1. In the following sections, we review the literature and further specify our rationale and hypotheses development for testing these associations.

### 3.1 Ethical leadership and safety voice

Research on the importance of ethical leadership has gained momentum over the past 15 years or so following a series of scandals (such as Enron), which highlighted poor moral conduct as a “root cause” of many organizational failings that had an adverse impact on employees and wider society (Brown & Treviño, 2006). In the present study, ethical leadership is broadly conceptualized in line with previous research as the “demonstration of normatively appropriate conduct through personal actions and interpersonal relationships, and the promotion of such conduct to followers through two‐way communication, reinforcement, and decision‐making” (Brown et al., 2005, p. 120). What may be considered “normatively appropriate conduct” in this sense can vary in form and emphasis depending on the context, but regarding health safety typically encompasses the core ethical principles autonomy (control by the individual), beneficence (do good), nonmaleficence (do no harm), justice (be fair), and fidelity (be true and faithful) (Beauchamp & Childress, 2001).

In keeping with these principles, the practical expression of ethical leadership is further understood to comprise two key dimensions known as the “moral person” dimension and the “moral manager” dimension. The moral person dimension reflects the demonstration of qualities such as “trustworthiness,” “integrity,” and “care for others” and is associated with a “transformational” leadership style (Kuenzi et al., 2020). Following perspectives such as social learning theory, it is thought that people tend to seek ethical guidance from attractive exemplars (Jordan et al., 2013), and due to the prestige and seniority they hold in the workplace, organizational leaders can accordingly provide a source of ethical role modeling (Bandura, 1986; Brown & Treviño, 2006). This means that leaders will display appropriate moral attitudes and conduct in the workplace, which may then serve as an exemplar from which employees can observe, learn from, and emulate in their own outlooks and behavior. Leaders attributed with high ethical standards may show that they care for their employees through mentoring, being inclusive, and identifying with colleagues and their respective concerns (Haslam et al., 2021). A shared sense of identity has also been shown to enhance perceived legitimacy and strengthen the collective resolve required of group members to comply with prescribed safety measures even when it requires personal sacrifice or imposes a large individual cost (Haslam et al., 2021; Wardman, 2020). Employees are thereby more likely to emulate desired attributes by voluntarily engaging in behavior that helps to improve their workplace safety environment as they trust both that they will be listened to and respected, and that displaying such behavior is normatively valued by leaders within their organization (Conchie & Burns, 2008).

The “moral manager” dimension of ethical leadership focuses on the establishment of governance mechanisms for promoting ethical conduct in the workplace and is more closely associated with a “transactional” leadership approach to influencing the behavior of other organizational members (Mayer et al., 2012). For instance, leaders can explicitly set standards for ethical conduct and hold their employees accountable for their (un)ethical actions by rewarding “good” moral conduct and penalizing “bad” moral conduct (Bhal & Dadhich, 2011). They can also set out procedures and processes that support fairness and inclusivity. In practice, ethical leadership can often comprise a combination of each moral dimension and incorporate both transformational and transactional leadership approaches. Workplace safety behavior will therefore be shaped through a mixture of mechanisms such as role modeling and governance processes that inspire and encourage the promotion of positive organizational relations, such as transparency and open two‐way communication amongst managers and employees (Kalshoven et al., 2011).

In turn, the concept of “safety voice” has been used to refer to communicative behaviors broadly undertaken by employees that aim to improve knowledge of risk and identify barriers to safety within an organizational environment (cf Conchie et al., 2012; Noort et al., 2019). As a type of extra‐role behavior, the display of safety voice is also considered to go above and beyond what is necessarily specified in formal contracts or legal requirements (LePine & Van Dyne, 1998). Safety voice has accordingly been found to be an important driver of safety performance (Curcuruto et al., 2015; Li et al., 2017) with the character of social exchange process between organizational leaders and employees shown to be particularly important to its expression (Sherf et al., 2021; Tucker & Turner, 2015). For example, engaging in safety voice can create discomfort in the workplace as it is likely to include critical observations that may reflect badly on peers, supervisors, or managers. For these reasons, workers may face some concerns and setbacks when displaying safety voice, including retaliation (Collinson, 1999), hierarchical barriers (Weiss et al., 2018), and conflicts of interest (Okuyama et al., 2014). Consequently, without encouragement from leaders, employees are more likely to remain silent about issues that may later prove disruptive and so refrain from expressing any opposing views (Xu et al., 2015; Noort et al., 2021). In more extreme cases, the continued absence of support, or even explicit discouragement of safety voice may lead employees to engage in “whistleblowing”. This occurs when individuals feel compelled to make wider appeals for help to parties outside their organization if they are left frustrated by internal responses and what they perceive to be a lax attitude to risk in their workplace (Noort et al., 2019; Zhang et al., 2019).

Ethical leaders may alternatively attempt to provide “safe environments” within organizations for open communication and welcome exchange of safety knowledge and ideas about risk management. Indeed, one of the premises of ethical leadership is to “provide followers with voice” (Brown et al., 2005, p. 120). Strong ethical leadership can therefore help to facilitate an organizational culture in which employees feel they can share risk‐related information and exchange knowledge and opinions with colleagues and managers without fear or favor because they know that their feedback will be received and used constructively to inform and support improvements to organizational processes and systems (Kuenzi et al., 2020). Workers may accordingly be surer of what to expect from their managers if they speak up about their workplace safety concerns than in the absence of ethical leadership where they may be deterred from doing so (Avey et al., 2012; Chen & Hou, 2016). Therefore, we argue that ethical leadership would have a facilitative role in supporting employee safety voice.

Hypothesis 1: Ethical leadership in the workplace is positively related to employee safety voice.

### 3.2 The mediating role of risk perception

While there is no universally agreed definition of risk perception, it is commonly conceived in academic literature to refer to the content and/or processes underlying people's appraisals of risky objects, situations, relations, and activities (Siegrist & Árvai, 2020; Slovic et al., 2004; Wilson et al., 2019). Understood in this broad sense, risk perception can be understood to reflect both analytical judgements and beliefs about the likelihood and severity of harm, as well as affective processes and emotional responses that shape these evaluations (Slovic et al., 2004; Walpole & Wilson, 2021; Wardman, 2006). How people perceive risk is considered important largely due to its association with behavioral decision making in the face of uncertainty across everyday life, as well as regarding extreme and rare events (e.g., Kahneman & Tversky, 1979; Wardman & Bouder, 2022). This makes it applicable to a wide variety of health, safety, financial, and environmental contexts, among others (Smith & Mayer, 2018). Risk perception also helps to shape people's behavior with respect to such matters as acceptance of environmental hazards (Grasmück & Scholz, 2005), sustainable behavior intentions (Spence et al., 2012), and environmental collaboration (Toma & Mathijs, 2007).

The perception of risk relating to potential harms and dangers in the workplace has likewise received considerable scholarly attention across wide ranging domains, including strategic management (e.g., Benischke et al., 2019), occupational health (e.g., Leiter et al., 2009), and safety (e.g., McDaniels et al., 1992). Albeit, variable findings are evident regarding the explanatory power of risk perception in shaping safety‐related behavior at work (Noort et al., 2019). Some studies have indicated that people's perception of risk has a positive impact on their safety‐related behavior in workplace settings (e.g., Goldberg et al., 1991); whereas other studies have suggested either negative (e.g., Christian et al., 2009) or no causal relationships (e.g., Rundmo, 1996). As with other areas of risk perception research, these conflicting findings are likely reflective of the wide spectrum of risk objects and the corresponding safety behaviors that occur within different contexts and in which numerous factors can be variably at play (Siegrist & Árvai, 2020). Therefore, while prior research can be highly informative, the emergence of a novel threat such as COVID‐19 typically merits new contextualized investigations to verify if, and how, it is perceived as a risk, and what, if any, safety‐related behavior is enacted, by different people, and why (Wardman & Lofstedt, 2020).

The emergence of the pandemic has understandably been followed by a deluge of risk perception studies that have largely confirmed the centrality of analytical and affective/associative appraisals of risk regarding COVID‐19, as well as the importance of other established factors such culture, trust, and political orientation, in shaping the ways people perceive and act upon this threat (e.g., Dryhurst et al., 2020; Siegrist et al., 2021; Wong et al., 2021). Nonetheless, studies addressing the perceived risks of contracting and transmitting the virus in the workplace are comparatively scarce. Looking to prior research, empirical evidence indicates that employees who are concerned about workplace safety are more likely to perceive those risks to be high and be self‐motivated to take proactive actions to mitigate them (Jones et al., 2017; Tam & Chan, 2018). Studies also further show that susceptibility to danger is an important factor for people when deciding to engage in safety‐related behaviors, such as communicating about risk when threatened (e.g., Rosenstock, 1974; Taylor & Snyder, 2017). As COVID‐19 is known to pose a direct threat to health for many people when contracted, this may further incentivize employees to engage in communicative actions to help mitigate the risk of coronavirus spreading at work (Siegrist et al., 2021).

Additionally, as a manager's conduct can give a strong indication of an organization's objectives, preferences, and concerns (Eisenberger et al., 1997), the acknowledgment of possible risks and encouragement from managers to communicating about them can help employees feel that their organization prioritizes safety over other performance indicators (DeJoy, 1996). This affirmation of organizational norms and expectations can then reduce psychological barriers to communicating about safety‐related concerns as employees may be less wary of causing disruptions (Wong et al., 2021). One key characteristic of ethical leadership is thus the encouragement of open discussion about risk issues of concern facing employees (Bavik et al., 2018; Cels, 2017) because ethical leaders recognize and are concerned about the risks others face (Wardman, 2020). In such situations as recognizing the need for COVID‐19 control measures, ethical leaders will accordingly impress upon workers the significance of the risk in question and facilitate a favorable environment for raising awareness and communicating safety concerns, thereby heightening risk perceptions and support for engaging in safety related behavior. We therefore predict that ethical leadership increases perception of COVID‐19 risk and that risk perception has a positive impact on safety voice in the workplace.

Hypothesis 2: Risk perception mediates the link between ethical leadership and safety voice.

### 3.3 The mediating role of ethical ambiguity regarding workplace decisions

Research shows that decision ambiguity amongst workers is associated with safety performance outcomes (e.g., Martínez‐Córcoles et al., 2014; Wang et al., 2018). Ethical ambiguity surrounding workplace decisions arises primarily from a perceived lack of information about job role and performance expectations, but also uncertainty about appropriate moral conduct when performing work duties (Breaugh & Colihan, 1994; Singh & Rhoads, 1991). For instance, when ambiguity is high people are more likely to set their own goals and work procedures that are at variance with formal guidelines and the behavior of other employees in the workplace (Yun et al., 2007). When ambiguity is low, on the other hand, workers can have an equally clear vision of how to align their behaviors with management expectations.

One antecedent of the level of ambiguity workers may perceive about their job role and performance is the frequency and quality of communication they share with their managers because this allows them to receive information and can gain direct answers to their questions (Dulebohn et al., 2012; Schwepker & Good, 2017). Similarly, employees who are involved in safety decisions will also perceive less ambiguity about appropriate conduct and what behaviors to perform in a given situation (Teas, 1983). In these regards, it is characteristic of ethical leadership, first, to maintain open communication, such that information is freely shared and employees have opportunities for open dialog with their managers, (Walumbwa et al., 2011); and second, to involve other members, such as by delegating decisions and consulting with those who are responsible for executing organizational strategies and operations on the front line (Thiel et al., 2018). These characteristics suggest that ethical leadership will thereby reduce ethical ambiguity for workers in organizations.

Research also suggests that the influence of leaders through such means as role modeling is likely to be stronger the context of COVID‐19 because when faced with external shocks employees rely more on steering signals by their leaders in order to deal with the inherent uncertainty that rare and unprecedented circumstances can bring to organizations (Crossan et al., 2008). This is not to say that leaders will necessarily act ethically under stress, this is often far from the case (Wardman, 2020), but rather that the actions of ethical leaders will more firmly reduce ethical ambiguity, which helps to ensure appropriate safety‐related behavior maintained by those in their charge.

Workers who are well‐informed about where managers stand on particular issues, and what their moral duty would be to fellow workers in their job role in difficult circumstances, are less likely to fear repercussions and feel more certainty that speaking up and sharing information about safety‐related matters is the right thing to do during risk and crisis events because they have a clearer understanding of what is expected of them when performing their jobs safely. We propose, therefore, that ethical ambiguity will be a mediating mechanism between ethical leadership and engagement in safety voice.

Hypothesis 3: Ethical ambiguity regarding workplace decisions mediates the relationship between ethical leadership and safety voice.

## 4 METHODS

### 4.1 Sample and data collection

Participants were recruited via a panel provider, Qualtrics, which made our study available to its pool of participants through its online platform. A total of 5304 individuals attempted to take part in the study until we reached a final sample size of 511 in 9 days. 3209 participants were screened out initially because they did not meet the specified criteria for participation, which were having a line manager, working at a commercial organization in full‐time employment, and with a minimum 1 year of work experience in their current organizations. Of the 2095 potential participants who passed the specified eligibility criteria, a further 1262 participants were screened out for careless responding. While 1196 failed at least one of the two attention filtering questions, 66 completed the study unfeasibly quickly. Finally, 322 participants dropped out before completing our study. The mean age of the final sample was 43.93 (SD = 10.70), with 54% of the participants being female. Participants had on average 23.13 years (SD = 11.47) of work experience and the average tenure in their current organization was 9.31 years (SD = 8.44).

We examined the likelihood of nonresponse bias in our sample by comparing the responses of early and late participants. The results show that there is no significant difference (*p* > 0.1). We further compared our final sample with the 322 participants who dropped out before the end of the survey in terms of work experience and industry. Again, we found no statistically significant differences between these groups. We conclude, therefore, that our final sample did not show any evidence of systematic bias.

### 4.2 Measurement of variables

The full set of the items is presented in Table 2.

#### 4.2.1 Ethical leadership

We measured ethical leadership using Brown et al.’s (2005) 10‐item measure. Responses ranged from *highly unlikely* (= 1) to *highly likely* (= 7). A sample item is “My line manager listens to what employees have to say.”

#### 4.2.2 Ethical ambiguity regarding workplace decisions

This construct was measured with Johlke & Duhan's (2000) seven‐point, three‐item measure. Responses ranged from *strongly disagree* (= 1) to *strongly agree* (= 7). A sample item is “I am certain what I am expected to do if I find others are behaving unethically.”

#### 4.2.3 Risk perception

This construct was measured with a four‐point, two‐item measure adapted from Spence et al. (2012). Responses ranged from *not serious at all* (= 1) to *very serious* (= 4). A sample item is “How serious a problem do you think coronavirus is?”

#### 4.2.4 Safety voice

We measured safety voice regarding COVID‐19 with five items adapted from Tucker et al. (2008). Responses range from *very unlikely* (= 1) to *very likely* (= 4). A sample item is “I suggest changes to company procedures to address coronavirus.”

#### 4.2.5 Control variable

Similar to other studies investigating the impact of ethical leadership on employee behaviors (Chen & Hou, 2016; Huang & Paterson, 2017; Young et al., 2021), we used participants’ educational attainment levels as a control variable.

## 5 RESULTS

### 5.1 Reliability and validity

Table 1 presents descriptive statistics, correlations, and reliabilities. All measures had Cronbach's alpha coefficients above the recommended 0.70 cut‐off (Nunnally, 1978), demonstrating acceptable internal consistency for all constructs.

**TABLE 1:** Descriptive statistics and intercorrelations

	**1**	**2**	**3**	**4**	**5**	**6**	**Mean**	**SD**
**1. Tenure**	–						9.31	8.44
**2. Education**	−0.18**	–					4.38	1.41
**3. Ethical leadership**	−0.09*	0.05	**0.96**				5.28	1.36
**4. Risk perception**	0.02	0.12**	0.25**	**0.91**			3.06	0.91
**5. Ethical ambiguity regarding work decisions**	−0.07	0.06	0.63**	0.17**	**0.88**		5.56	1.27
**6. Safety voice**	−0.05	0.16**	0.23**	0.40**	0.29**	**0.76**	2.84	0.62

*Notes*: *N* = 511. Cronbach's alpha coefficients are presented in bold in the diagonal.

* *p* < 0.05.

** *p* < 0.01.

We assessed the convergent and discriminant validity of our measures using exploratory and confirmatory factor analyses. We first submitted measures to exploratory factor analysis using the direct oblimin rotation method. Table 2 presents the results of the exploratory factor analysis results, which indicates that measures have acceptable convergent and discriminant validity. We further examined the discriminant validity of the constructs using the average variance extracted (AVE) measures obtained from confirmatory factor analysis. All AVEs were above the threshold of 0.50 (Fornell & Larcker, 1981), providing evidence for discriminant validity.

**TABLE 2:** Exploratory factor analyses results

**Factor and Items**	**F1**	**F2**	**F3**	**F4**
**F1: Ethical leadership**				
1. My line manager can be trusted.	**0.98**	0.00	−0.17	0.00
2. My line manager makes fair and balanced decisions.	**0.95**	−0.02	−0.04	−0.01
3. My line manager has the best interests of employees in mind.	**0.93**	−0.04	0.00	−0.01
4. My line manager sets an example of how to do things the right way in terms of ethics.	**0.92**	0.01	0.01	0.02
5. My line manager defines success not just by results but also the way that they are obtained	**0.88**	−0.01	0.03	0.06
6. When making decisions, my line manager asks “what is the right thing to do?”	**0.88**	0.03	0.01	0.00
7. My line manager listens to what employees have to say.	**0.85**	0.00	0.01	−0.02
8. My line manager discusses business ethics or values with employees.	**0.74**	0.06	0.08	−0.03
9. My line manager conducts his/her personal life in an ethical manner.	**0.70**	−0.02	0.12	0.00
10. My line manager disciplines employees who violate ethical standards.	**0.41**	0.00	0.26	−0.12
**F2: Safety voice**				
1. I intend to speak to key people in charge about coronavirus.	0.10	**0.84**	‐0.13	0.05
2. Suggest changes to company procedures to address coronavirus.	‐0.02	**0.83**	0.03	0.00
3. I intend to take part in a campaign about coronavirus.	0.05	**0.78**	‐0.02	0.08
4. I intent to seek more information about coronavirus.	‐0.08	**0.60**	0.09	‐0.10
5. I intend to discuss coronavirus with colleagues.	‐0.11	**0.44**	0.13	‐0.13
**F3: Ethical ambiguity regarding work decisions**				
1. I am certain how I should handle ethical issues in my job.	‐0.03	0.06	**0.90**	0.05
2. I am certain what I am expected to do if I find others are behaving unethically.	0.02	0.01	**0.90**	0.02
3. In my job, I am certain of the ethical conduct my supervisor expects of me.	0.22	‐0.05	**0.75**	0.01
**F4: Risk perception**				
How serious a problem do you think coronavirus is?	‐0.01	0.05	0.03	**0.98**
How concerned, if at all, are you about coronavirus, sometimes referred to as ‘COVID‐19′?	‐0.02	‐0.09	0.03	**0.91**
Eigenvalues	8.79	2.9	1.29	1.21
Total variance explained by each factor	43.99	14.55	6.48	6.05
Cumulative variance explained by the factors	43.99	58.54	65.02	71.07

*Note*: *N* = 511. Bold is used to highlight the loading between an item and its respective scale/factor.

Discriminant validity of the proposed measurement model was also tested against alternative nested models. Accordingly, we first compared the proposed four‐factor model with two three‐factor models in which independent variable items and one of the mediator variable items were specified to load into a single factor (Model 2 and 3). Then, we compared it with an alternative one‐factor model in which all items were specified to load into a one common factor. The results presented in Table 3 below show that the proposed model had good fit with data (*χ* ^2^/df = 3.41, CFI = 0.95, TLI = 0.94, RMSEA = 0.07). However, alternative nested models did not have adequate fit. Therefore, CFA results offer further support for the discriminant validity for the measures used in this study.

**TABLE 3:** Confirmatory tests of the discriminant validity of the measures

**Model name**	***χ* ^2^(df)**	**Δ*χ* ^2^(Δdf)**	***p***	***χ* ^2^/df**	**CFI**	**TLI**	**RMSEA**
1: Proposed 4‐factor model	569.87 (165)			3.41	0.95	0.94	0.07
Alternative 3‐factor models							
2: Merging ethical leadership and risk perception	1256.24(167)	686.37(3)	0.00	7.52	0.85	0.84	0.11
3: Merging ethical leadership and ethical ambiguity regarding work decisions	1043.592(167)	473.72(3)	0.00	6.25	0.88	0.87	0.1
4: Alternative 1‐factor model	2257.97 (170)	1688.10(6)	0.00	13.28	0.73	0.7	0.16

*Note*: *N* = 511. CFI (comparative fit index), TLI (Tucker‐Lewis index), RMSEA (root mean square error of approximation), SRMR (standardized root mean square residual).

### 5.2 Common method bias

We minimized the potential risk of common method bias by taking into account several research data collection considerations. First, we informed participants that responses would be kept anonymous and that data collected would be presented in an aggregated format in order to ensure anonymity (Chang et al., 2010; Podsakoff et al., 2012). Second, we randomized items within constructs and separated independent and dependent variables to minimize the effects of hypothesis guessing.

We also performed post‐hoc statistical analyses to test common methods bias. Accordingly, we first ran Harman's single factor test to check whether a single factor consisting of all items in the study explains much of the variance. The results suggest that no single common factor accounted for the majority of the variance. Second, we ran a marker variable test following Lindell & Whitney (2001). We used work experience as a marker variable due to the fact that there is no theoretical basis for work experience to relate to the substantive variables of this study. Accordingly, we identified the lowest correlation between the marker variable and the substantive variable (i.e., *r* = 0.01 between work experience and risk perception). We subtracted this estimate from each correlation between substantive variables and divided adjusted correlations with 1 minus this estimate. Results show that absolute difference between unadjusted and common method bias adjusted correlations were relatively small, ranging between 0.01 and 0.05. Based on these results, we conclude that common method bias does not pose a serious problem in our study.

We also examined multicollinearity among measures. As shown in Table 2, all pairwise correlations were less than the threshold of 0.70, values greater than which indicates a higher risk of collinearity (Tabachnick & Fidell, 2013). Results show that all tolerance were above 0.60 and none of the variance inflation factor (VIF) values were greater than 1.7, considerably lower than the recommended cut‐off value of 10 (Hair et al., 2010). Therefore, multicollinearity is not deemed to pose a serious threat in our study.

### 5.3 Hypothesis testing

We used hierarchical regression to test the hypotheses. Table 4 reports the results of the regression analysis which includes five models. Model 1 consists of control variables (i.e., education and tenure) and ethical leadership. Models 2 and 3 are used to test the mediation effect of risk perception on the relationship between ethical leadership and safety voice. While Model 2 regresses risk perception on ethical leadership and control variables, Model 3 regresses safety voice on risk perception, ethical leadership, and control variables. Similarly, Models 4 and 5 are used to test the mediation effect of ambiguity regarding ethical decisions on the relationship between ethical leadership and safety voice. While Model 4 regresses ambiguity regarding decisions on ethical leadership and control variables, Model 5 regresses safety voice on ambiguity regarding ethical decisions, ethical leadership, and control variables.

**TABLE 4:** Hierarchical regression results

	**Model 1: Safety voice**			**Model 2: Risk perception**			**Model 3: Safety voice**			**Model 4: Ambiguity regrding ethical decisions**			**Model 5: Safety voice**		
	B	SD	*t*	B	SD	*t*	Beta	SD	*t*	B	SD	*t*	B	SD	*t*
Intercept	2.00	0.13	15.23**	2.25	0.2	11.49**	1.45	0.14	10.54**	2.39	0.23	10.22**	1.74	0.15	11.34**
Education	0.07	0.02	3.60**	0.11	0.03	2.58**	0.05	0.02	2.83**	0.02	0.03	0.74	0.06	0.02	3.52**
Ethical leadership	0.1	0.02	5.23**	0.1	0.03	3.20**	0.08	0.02	4.31**	0.58	0.03	18.17**	0.04	0.03	1.45
Risk perception							0.25	0.03	8.85**						
Ethical ambiguity regarding work decisions													0.11	0.03	4.28**
R‐squared	0.08			0.04			0.20			0.39			0.11		

*Notes*: *N* = 511.

* *p* < 0.05.

** *p* < 0.01.

Supporting our first hypothesis, Model 1 in Table 4 shows that ethical leadership has a significant positive effect on safety voice regarding COVID‐19 (*β* = 0.1, *p* < 0.01). Hypothesis 2, which proposes that risk perception mediates the relationship between ethical leadership and safety voice, was also supported. Model 2 reveals that ethical leadership has a positive impact on risk perception (*β* = 0.15, *p* < 0.01). Additionally, Model 3 reveals that risk perception has a positive impact on safety voice when ethical leadership is controlled (*β* = 0.25, *p* < 0.01). Further, we analyzed the indirect effect of ethical leadership through a bias‐corrected bootstrap interval based on 5000 samples (Hayes, 2013). The indirect effect was significant (*p* < 0.01) and the 95% bootstrap confidence interval did not contain zero (LCI = 0.02, UCI = 0.06), providing strong support for Hypothesis 2.

Similarly, Hypothesis 3, which proposes that ambiguity regarding ethical decisions mediates the relationship between ethical leadership and safety voice, was supported. Model 4 in Table 4 reveals that ethical leadership has a positive impact on reducing ambiguity regarding ethical decisions (*β* = 0.58, *p* < 0.01). Additionally, Model 5 reveals that ambiguity regarding ethical decision has a significant impact on safety voice regarding COVID‐19 when ethical leadership is controlled (*β* = 0.11, *p* < 0.01). Further, we analyzed the indirect effect of ethical leadership through a bias‐corrected bootstrap interval based on 5000 samples. The indirect effect was significant (*p* < 0.01) and the 95% bootstrap confidence interval did not contain zero (LCI = 0.04, UCI = 0.10), providing strong support for Hypothesis 3.

**FIGURE 2:**
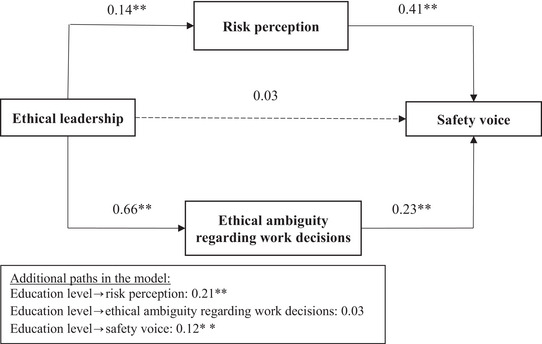
Results of structural equation model. Notes: *N* = 511, ^*^ *p* < 0.05, ^**^ *p* < 0.01

We also tested our full model with structural equation modeling (SEM) using AMOS 28.0. The results of the model are presented in Figure 2. Among the control variables, education was significantly related to risk perception (*β* = 0.21, *p* < 0.01) and safety voice (*β* = 0.12, *p* < 0.01). Examination of path coefficients reveals that risk perception and ambiguity regarding ethical decisions mediate the impact of ethical leadership on safety voice. The path coefficient between ethical leadership and safety voice was not significant in the full model, which indicates that the impact of ethical leadership on safety voice is fully mediated by risk perception and ethical ambiguity regarding work decisions.

## 6 DISCUSSION

This study examined how ethical leadership has affected safety voice during the COVID‐19 pandemic, a crisis which has been punctuated throughout by conduct failures by leaders and senior officials in public and private sector organizations. Building on previous research showing that leadership style (Braumann et al., 2020) and safety voice (Noort et al., 2019) both respectively contribute to improving organizational safety (Curcuruto et al., 2015), our findings reveal that ethical leadership plays an important role in supporting these processes by having a positive effect on increasing risk perception and reducing ethical ambiguity regarding decisions in the workplace. Our study thereby provides several contributions to the wider literature on leadership, risk communication, and risk management, and carries some interesting theoretical and practical implications, which are now discussed in turn.

### 6.1 Theoretical implications

Firstly, in theoretical terms, our study extends current knowledge by elaborating key mechanisms by which ethical leadership can support safety voice. Previous studies indicate that good moral conduct by leaders is important because it helps to set expectations of acceptable risk taking and corresponding safety‐related behavior (Braumann et al., 2020; Chen & Hou, 2016; Stollberger et al., 2020). Conversely, poor conduct by leaders, such as ignoring risk or failing to abide by safety rules, can undermine wider adherence to advised safety measures by sowing doubt, division and resentment (Reicher, 2021). Yet, despite the growing interest shown toward the relationship between leadership style and workplace safety (Inness et al., 2010; Mullen et al., 2011), research on ethical leadership within an organizational context is scarce (Chughtai, 2015; Walumbwa & Schaubroeck, 2009). Our research provides a significant step toward filling this void by showing that ethical leadership is also an important component to promoting safety in the workplace by encouraging worker engagement in safety voice.

Secondly, while leadership style is proven to have an important influence on safety behaviors (Chughtai, 2015; Hu et al., 2018), less is known about the intervening mechanisms through which ethical leadership affects safety voice. Ethical leaders characteristically give clear signals to employees about the importance of following ethical codes of conduct in the workplace (Chen & Hou, 2016), and support communication between workers and managers (Noort et al., 2019). Building on work showing ethical ambiguity can affect safety behavior (Parboteeah & Kapp, 2008; Richter & Koch, 2004), our study extends this research by showing ethical ambiguity can mediate the relationship between ethical leadership and safety voice. Particularly, ethical leadership can contribute to reducing ethical ambiguity which encourages the expression of safety voice.

Thirdly, our study also contributes to current understandings of the role of risk perception in workplace safety. Previous studies have largely focused on well‐established risks with tangible consequences, such as everyday workplace accidents (Arezes & Miguel, 2008). We provide additional empirical evidence suggesting that risk perception also plays an important role in mediating relations between ethical leadership and safety voice in organizations when the risk of concern is novel, uncertain, and complex, as demonstrated in the case of COVID‐19. Particularly, ethical leadership is associated with an increase in risk perception which can encourage safety voice. These findings therefore underscore the importance of ethical leadership for ensuring that risks are understood and acted upon in the interests of organizational members across a wider variety of work conditions and circumstances, including both day‐to‐day risk management concerns as well as crisis situations.

### 6.2 Practical and policy implications

Our findings provide clear evidence that ethical leadership is of key importance for promoting safety voice within organizations. Ethical leaders are understood to care about employee's health and well‐being so are more likely to help facilitate working cultures and environments that promote the exchange of risk‐related knowledge and workplace safety concerns in order to better understand and mitigate health and safety threats and hazards (Tucker at al., 2008). Following our findings, we recommend that organizations accordingly foster ethical leadership and safety voice in several ways which can help to support ethical conduct and safety behavior (Brown et al., 2005). First, ethical considerations can be incorporated as key hiring criteria during recruitment to help ensure the appointment of ethically attentive managers and personnel. Second, appraisal processes for managers can explicitly evaluate and reward ethical conduct. Thirdly, as our findings show that safety voice behavior can be enhanced by reducing employees’ perceived ethical ambiguity for workplace decisions, organizations can enact ethics training programs and mentoring to help workers identify, reduce, and resolve ethical ambiguities when difficult decisions are encountered. Organizations may also create a code of ethics and incorporate this into a mission statement that conveys the standards and expectations of ethical conduct an organization aspires to uphold. Fourthly, managers can also make transparent how ethical considerations should figure in day‐to‐day strategies, operations, and decision‐making processes to help employees to understand the ethical basis of evaluating and choosing between alternative courses of action that they are likely to be confronted with, as well as general principles to follow when faced with new situations. Fifthly, as evidenced elsewhere, understanding and appreciating both the direct and indirect impacts of risks such as COVID‐19 can contribute to risk concerns and in turn support their mitigating behaviors (Dryhurst et al., 2020; Siegrist et al., 2021). Our findings with regards to COVID‐19 reveal that heightened risk perception is associated with improved levels of safety voice behavior. This therefore supports the argument that managers should raise awareness of risks and signal how leaders within the organization appraise those risks, along with their views on the benefits of communication for employees verses the possible consequences that failing to recognize and act on such risk might have on organizational members and their wider community.

### 6.3 Limitations and future research

This study is the first to our knowledge to directly examine the associations between ethical leadership and safety voice. However, future research can extend the model introduced in this study by considering the broader nomological network of ethical leadership and workplace safety in several additional ways. For instance, while we establish a link between ethical leadership and safety voice, we did not confirm that safety voice enhanced by ethical leadership would lead to better safety performance. Further research might accordingly investigate the impact on safety performance through the use of broader measures and objective indicators of safety‐related behaviors and outcomes. This might be applied both generally (e.g., safety participation and compliance, accident reports), and in relation to COVID‐19 specifically (e.g., cases of coronavirus in the workplace).

As mentioned above, the ethical leadership measure adopted in this study, and more generally in the extant literature, is concerned with employees’ evaluations of their managers’ characteristics and actions (Curcuruto et al., 2016; Noort et al., 2019). This invites consideration of at least two further possible extensions to our research. Firstly, prior studies of group psychology have indicated that people who are seen as ‘ingroup’ members enjoy higher trust and more influence than someone who is seen as ‘outgroup’ (Reicher, 2021). In this prior work, leadership is understood to be made possible by the way in which leaders have a shared sense of identity with followers who subsequently place greater trust in them (Reicher et al., 2005; Wardman, 2020). If the behavior of leaders is not in accordance with the norms and expectations of the ingroup, as when ethical conduct violations come to light, shared identity can be undermined meaning that leaders will no longer be seen as ingroup members resulting in them having less influence and trust among followers (Reicher et al., 2005). While we examined perceptions of ethical leadership by workers, we did not specifically examine their perceptions of shared identify with leaders or test levels of trust. Questions therefore remain concerning the impacts of ethical leadership on shared identity and trust, which could be empirically tested in further research.

Second, there is presently little empirical research investigating the antecedents and consequences of ethical leadership behaviors. Future studies could use (quasi) experimental methods for the direct measurement of factors contributing to ethical leadership behaviors and their impact on organizational outcomes. Studies might also consider including the measurement of broader leadership styles that have some moral component (e.g., transformational leadership), which would add further incremental validity and help to further delineate the conceptual distinctiveness of ethical leadership.

In this study, we incorporate a broad measure of safety voice following a well‐established conventional formulation, however, recent research has also begun to elucidate the multitudinous forms that safety voice can take, including both within (Bazzoli & Curcuruto, 2021), and outside organizations (Noort et al., 2019). Future research could incorporate these recent considerations to further distinguish the relative importance of different mediums and processes of safety voice, along with how they may specifically relate to the associations identified in our study.

We also note that this study was conducted in the early phase of the pandemic, but did not track changes over time. Our study was also confined to data collection among companies within the United Kingdom, and so was not able to provide comparisons across different regions, cultures, and sectors, which have been shown to be important in other studies investigating risk perceptions and behavior in response to the COVID‐19 pandemic (e.g., Dryhurst et al., 2020). Further research might then also examine the relations of ethical leadership and safety voice accounting for these wider considerations. Lastly, our results are based on cross‐sectional data. Future research might employ a longitudinal research design to provide further evidence for the causality among variables used.

## 7 CONCLUSIONS

The display of questionable integrity by leaders, as observed through flagrant safety breaches across all sectors ranging from the factory floor to the head of government, is both regrettable and feared to undermine collective public health efforts to keep workplaces “COVID‐secure” (Reicher, 2021; Reicher et al., 2021). The possible consequences of making light of health risks and safety measures put in place to mitigate them are an increased risk of virus outbreaks harming workers and their wider communities, as well as the higher likelihood of future local lockdowns that impact on business viability amidst depreciations in worker morale and public and private sector reputations. Against this backdrop, the connection between ethical leadership and workplace safety remains an underexplored, yet important issue. In this study, we focused on ethical leadership due to questions and concerns about its importance in helping to ensure people respond effectively to threats focusing on the COVID‐19 pandemic. We find that ethical leaders are better equipped to promote safety behavior through such means as modeling good moral conduct and giving clear transparent guidance on expected moral standards to be upheld by followers in organizations when faced with risk. In light of these new findings, we suggest that there are ripe opportunities for future studies as there is still much to learn about how wider contextual influences might impact on interactions between ethical leadership and engagement in safety voice in different guises, and how this sustains varies over time within different organizational settings, and across different regions and cultures. We hope that our findings provide stimulation for further inquiry and critical discussion on ethical leadership, safety voice, and their relation to other forms of risk and crisis communication.
